# Dual inhibition of ABCE1 and LCP1 by microRNA-96 results in an additive effect in breast cancer mouse model

**DOI:** 10.18632/oncotarget.26747

**Published:** 2019-03-12

**Authors:** Nir Pillar, Avital Luba Polsky, Noam Shomron

**Affiliations:** ^1^ Sackler Faculty of Medicine, Tel Aviv University, Tel Aviv 69978, Israel

**Keywords:** breast cancer, ABCE1, LCP1, microRNA-96, metastasis

## Abstract

MicroRNAs (miRNAs) are short non-coding RNAs that regulate the expression of target genes at the post-transcriptional level. Each miRNA can modulate multiple genes and, as a result, a single miRNA may have a profound effect on a specific biological pathway consisting of several of its target genes. Recent studies have indicated that specific miRNA signatures are correlated with tumor aggressiveness and clinical outcome in breast cancer. We previously demonstrated that miR-96 has a suppressive effect on breast cancer aggressiveness and that this effect was mediated by ABCE1 gene regulation. In this study we investigated whether other miR-96 regulated genes can enhance ABCE1’s anti-cancer effects. We identified one such gene – LCP1 – and proved its negative effect on breast cancer progression. Interestingly, dual inhibition of ABCE1 and LCP1 resulted in an additive effect on cancer cell migration, invasion, and proliferation. Furthermore, *in vivo* analysis of dual ABCE1 and LCP1 knockdown resulted in significant tumor growth inhibition, decreased metastatic activity, and contributed to survival compared to either gene, separately. This indicates that the combined downregulation of two miR-96 gene targets has an additive effect on reducing cancer aggressiveness. Overall, our work supports seeking more than one target in miRNA-based studies in order to enhance functional effects and better characterize the miRNA wide-spread activity.

## INTRODUCTION

Breast cancer is a major cause of cancer morbidity and mortality in women worldwide [1–3]. Controlling metastasis—the main cause of breast cancer patient deaths—poses the greatest clinical, therapeutic challenge in this disease. It is, thus, necessary to develop more effective methods of metastasis prevention and treatment in order to increase survival. For decades, cancer was perceived as a disease of the genome, predominantly resulting from mutations in key genes. However, myriad studies over the last twenty years have demonstrated that epigenetic changes are associated with almost every step of tumor development and progression [4]. Additionally, it has become increasingly evident that epigenetic changes play a key role in the metastatic process [5]. miRNAs are non-coding small RNAs (∼22nt) that negatively regulate gene expression and are highly associated with tumorigenicity, invasion, and metastasis [6–8]. Each miRNA can regulate multiple genes that act in concert on the same biological pathway and considerably influence the pathway’s function [9, 10]. We recently identified microRNA-96 (miR-96) as a tumor suppressor gene and validated its effect *in vitro* and *in vivo*. Using gene expression analysis, we identified ABCE1 as a major miR-96 target and confirmed its effect on metastasis formation [11]. In this study, we asked whether ABCE1 acts alone or in concert with other genes. We identified one such gene, L-Plastin (LCP1), and assessed its effect on breast cancer progression. We showed that LCP1 has onco-suppressive effects *in vitro* and *in vivo*. We demonstrated that mutual LCP1 and ABCE1 reduction has an additive effect on cancer cell aggressiveness *in vitro* and *in vivo*, further supporting the one miRNA-multiple targets theory.

## RESULTS

### miR-96-target gene selection

**Figure 1:**
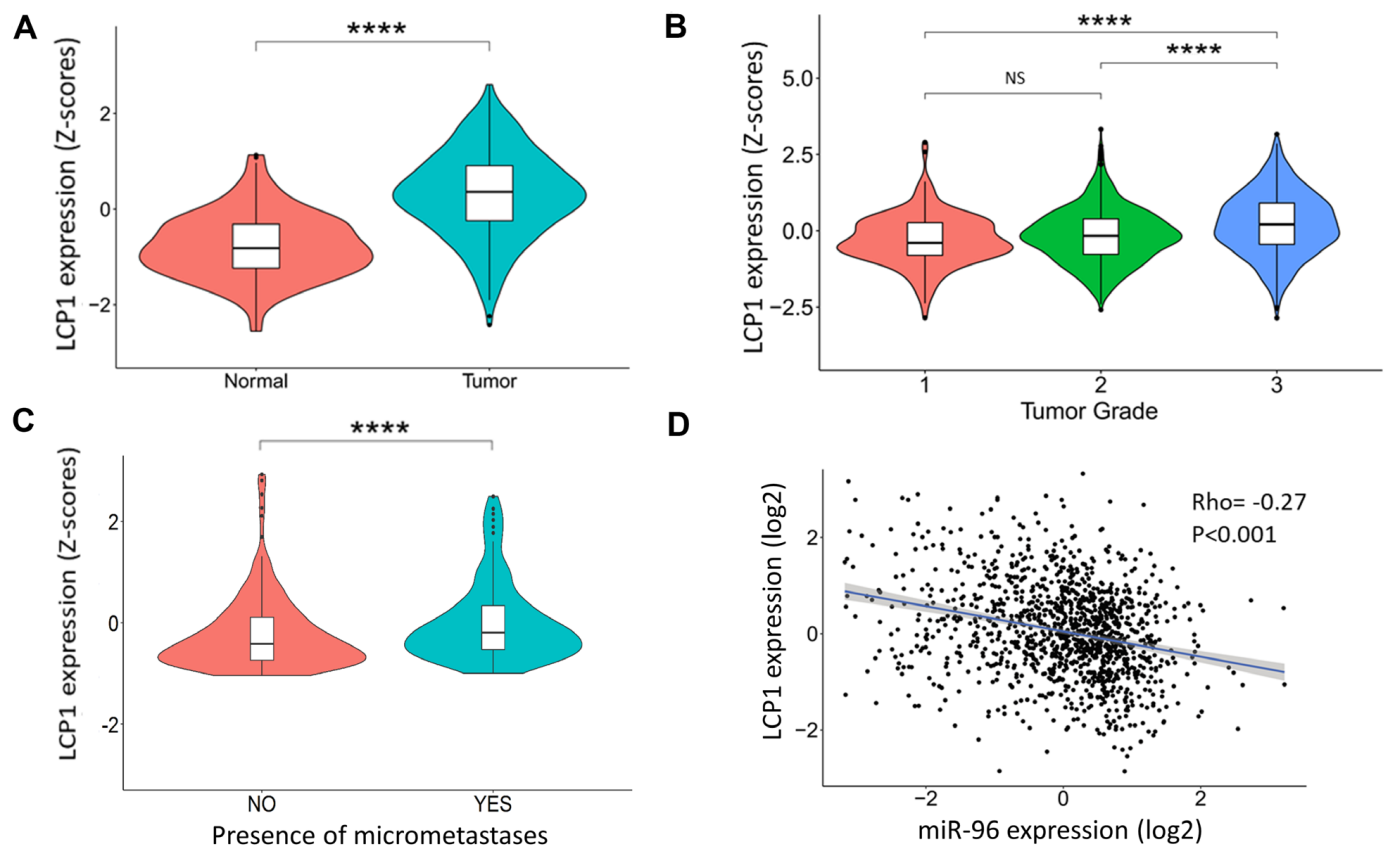
LCP1 gene expression in human breast cancer tissues (**A**) LCP1 gene expression in breast tumors and normal breast tissue. A significant increase in LCP1 expression is seen in cancerous tissues. Data was obtained from MERAV [37]. (**B**) LCP1 expression is directly correlated with tumor grade and (**C**) with the presence of micrometastases. Data was obtained from cBioPortal [38]. (**D**) Inverse correlation of LCP1 and miR-96 expression was noted in transcriptomic analysis of human breast tumors. Data was obtained from cBioPortal [38]. Data in violin plots is presented as Z-scores ± SEM. Correlation was calculated using Spearman’s rank correlation coefficient. ^****^p < 0.001. NS-not significant.

To test whether ABCE1 acts alone or in concert with other miR-96 target genes, we referred to our previously published list of twelve breast cancer-associated genes predicted to be regulated by miR-96 [11]. One of the hallmarks of the metastatic process is altered cellular motility [12], a complex process requiring dramatic remodeling of the cell cytoskeleton. Because accumulating evidence indicates that ABCE1 plays an important role in cytoskeleton regulation [13–15], PubMed curation was used to filter the remaining eleven genes (excluding ABCE1) by their association with cytoskeleton organization. We found that LCP1 is an actin binding protein that participates in cytoskeleton remodeling [16–18]. LCP1 has a median expression in non-diseased human breast compared to other tissues (Supplementary Figure 1, data analyzed using the GTEx project database [19]). However, LCP1 expression levels significantly increase in breast tumors (Figure 1A), are directly correlated with tumor severity (Figure 1B) and lymph node micrometastases presence (Figure 1C). LCP1 is inversely correlated with miR-96 expression in breast tumors (Figure 1D), suggesting LCP1’s role as a tumor-associated gene that is regulated by miR-96. Therefore, we proceeded to explore the role of LCP1 in breast cancer metastasis formation and, especially, the additive effect of LCP1 and ABCE1 on this process.

### miR-96 directly targets LCP1 and downregulates its expression

**Figure 2:**
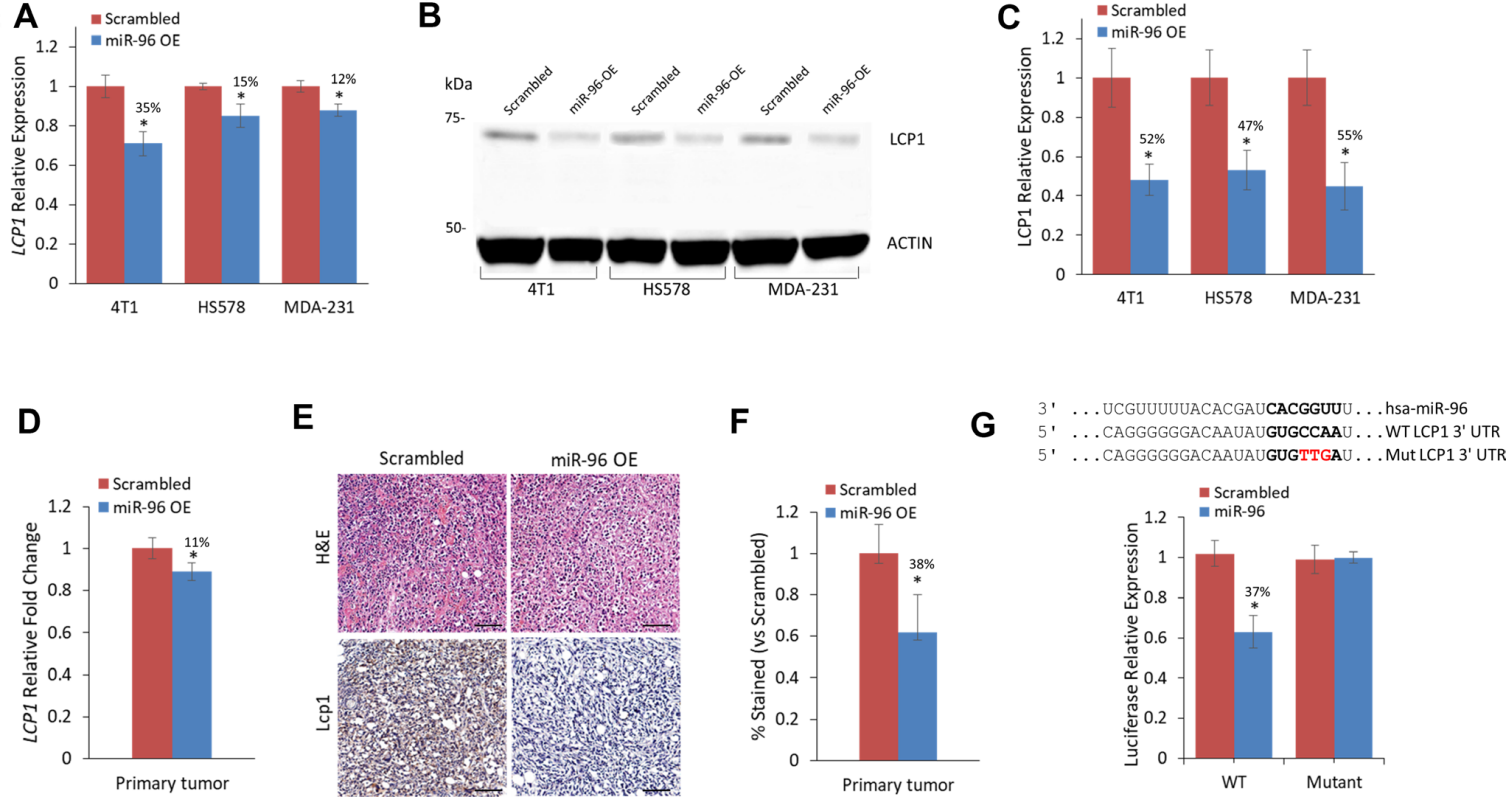
LCP1 gene and protein expression are reduced in direct response to miR-96 overexpression (**A**) *LCP1* expression levels in 4T1, HS578, and MDA-231 cell lines expressing miR-96 OE or scrambled control. *LCP1* expression is reduced in all cell lines with miR-96 OE. (**B**) Western blot of LCP1 in breast cancer cell lines and (**C**) calculated LCP1 protein expression in cell lines with miR-96 OE or scrambled control. In all cell lines, LCP1 expression is twofold decreased in miR-96 OE cells compared to scrambled control. (**D**) LCP1 in primary tumors of mice injected with miR-96 overexpressing or scrambled control 4T1 cells. LCP1 and miR-96 expression levels are inversely correlated (**E**) H&E and immunohistochemistry for Lcp1 of resected murine primary breast tumors. Scale bars represent 50 µm (**F**) Calculated difference in Lcp1 immunohistochemical staining between miR-96 OE or scrambled control. Reduced Lcp1 staining is seen in miR-96 OE compared to Scrambled. (**G**) Luciferase binding assay for miR-96 and Lcp1. Predicted binding site (indicated by bold letters) for hsa-miR-96 on the LCP1 3′-UTR. Wild type (WT) and mutant (Mut) miR-96 binding sites are presented. Red nucleotides represent the three mutated nucleotides in the miR-96 seed binding site. Luciferase activity 24 h following co-transfection of HeLa cells with hsa-miR-96 and LCP1 WT or Mut 3′-UTR construct. Significant decrease in Luciferase activity was seen when transfected with LCP1 WT but not with 3′-UTR Mut. Data are presented as mean ± SEM. ^*^p < 0.05, ^**^p < 0.01.

To evaluate LCP1 as a miR-96 target *in vitro* and *in vivo*, we examined its expression in 4T1, MDA-231, and HS578 breast cancer cells that overexpress miR-96 or scrambled miRNA. Previously, we assessed ABCE1 as a miR-96 target *in vitro* using total RNA and protein from cells overexpressing miR-96 or scrambled miRNA [11]. The same total RNA and protein extracts were used in this study to evaluate LCP1 expression on both the RNA and protein levels. LCP1 RNA expression in miR-96 overexpressing (OE) cells was reduced across all cell lines (Figure 2A). Western blot analysis of LCP1 in 4T1, HS578, and MDA-231 revealed a reduction of more than 50% in LCP1 protein expression in all miR-96 OE cells compared to scrambled (Figure 2B and 2C).

Next, we assessed *Lcp1* expression in mouse primary tumors generated from miR-96 OE or scrambled 4T1 cells, and noted a significant mean decrease of 10-15% in tumors generated from miR-96 OE cells (Figure 2D). Immunohistochemical analysis further corroborated this trend, with a 40% mean decrease in LCP1 expression in miR-96 OE primary tumors (Figure 2E and 2F).

To assess whether miR-96 directly regulates LCP1, we conducted luciferase reporter assays on HEK293 and HeLa cells co-transfected with miR-96, and WT or mutant LCP1 (Figure 2G). A significant reduction in luciferase activity was observed in cells that received WT LCP1 compared with those that received the mutated construct, indicating that miR-96 directly regulates LCP1 expression.

### Dual inhibition of ABCE1 and LCP1 reduces cancer cell migration, invasion, and proliferation

**Figure 3:**
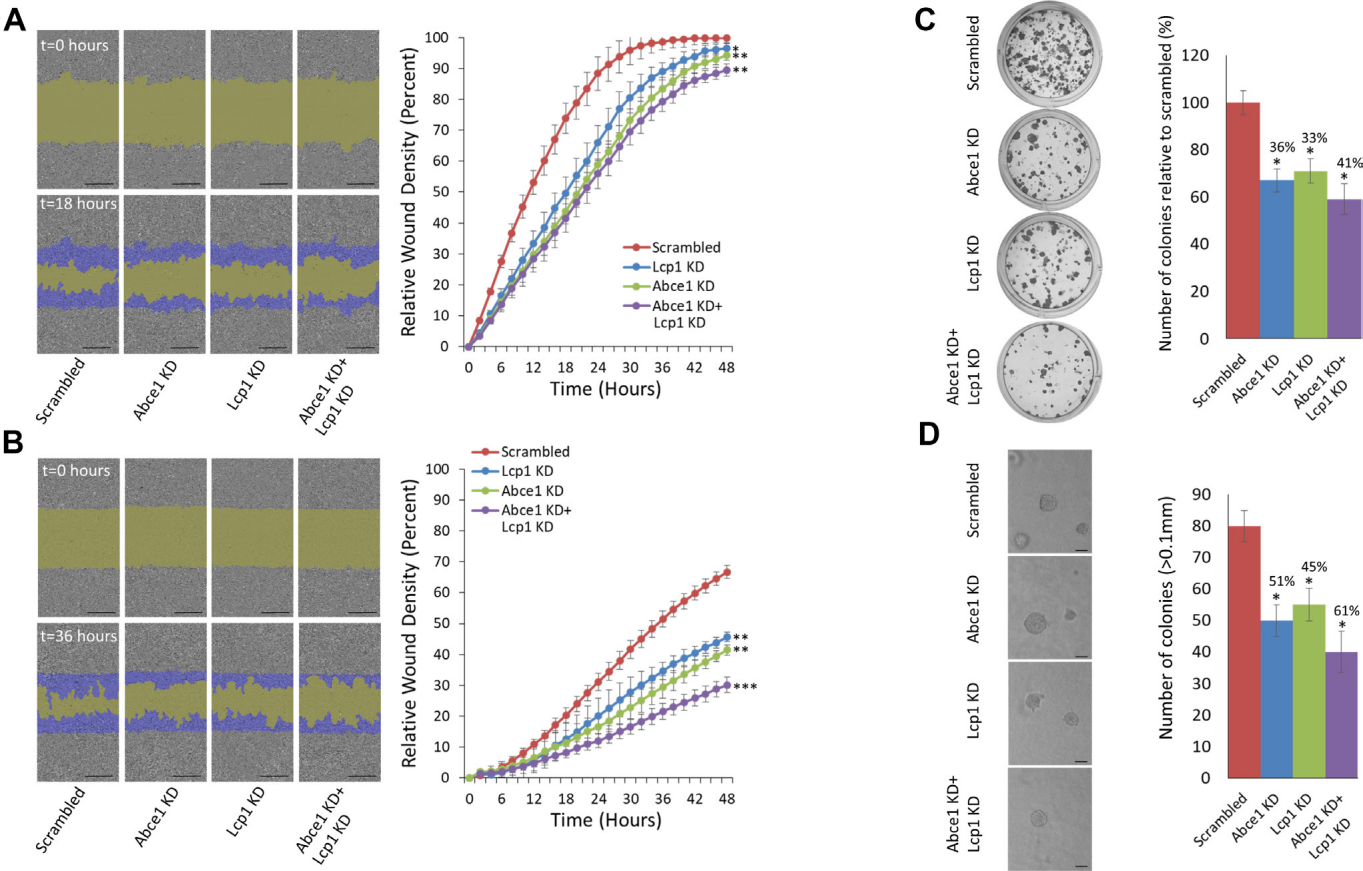
Simultaneous knockdown of LCP1 and ABCE1 has an additive effect on reduction of migration and invasion of 4T1 breast cancer cells (**A**) Representative images of a migration assay (left) and mean scratch area closure over time (right). The upper row represents time 0 and the bottom row represents 18 hours post-scratch. HS578 cells (expressing scrambled, ABCE1 KD, LCP1 KD, or ABCE1 KD + LCP1 KD) are shown in gray, the green area represents the scratch, and migrating cells are indicated in dark blue. The migration assay mean scratch area closure over time reveals a delay in the rate of migration of cells that underexpress ABCE1, LCP1 or both compared to the scrambled control. Data for each time point is presented as mean ± SEM ^*^p < 0.05 ^**^p < 0.01. Scale bars represent 300 µm. (**B**) Representative images of an invasion assay (left) and mean scratch area closure over time (right). The upper image represents time 0 and the bottom image represents 36 hours post-scratch. Invading HS578 cells (expressing scrambled, ABCE1 KD, LCP1 KD, or ABCE1 KD + LCP1 KD) are indicated in dark blue. The invasion assay mean scratch area closure over time demonstrates slower invasion rates of cells with LCP1 KD compared to Scrambled and an additive effect of dual ABCE1 and LCP1 KD compared to knockdown of each gene individually. Data for each time point is presented as mean ± SEM ^**^p < 0.01 ^***^p < 0.005. (**C**) Representative images (left) and quantification (right) of colony formation assays in 4T1 cells. (**D**) Representative images (left) and quantification (right) of colony numbers from anchorage-independent growth assays in 4T1 cells. Colonies were counted at 10× magnification using a phase contrast microscope. Only colonies larger than 0.1 mm in diameter were counted. Scale bars represent 200 µm. Both assays show that knockdown of either ABCE1 or LCP1 reduces colony formation, and simultaneous knockdown of both genes further reduces colony formation. Data are presented as mean ± SEM. ^*^p < 0.05.

To examine the effect of LCP1 knock down (KD) and dual ABCE1+ LCP1 KD on cell migration, we used shRNAs to stably underexpress ABCE1, LCP1, ABCE1+ LCP1, or scrambled in HS578 human breast cancer cells and 4T1 murine breast carcinoma cells. We validated the shRNAs effect on the gene and protein level (Supplementary Figure 2). Next, we conducted a scratch wound migration assay using the IncuCyte Live Cell Imaging System (Sartorius). A significant reduction in cell migration was observed in all study groups compared to the scrambled control (Figure 3A). Interestingly, dual inhibition of ABCE1 and LCP1 resulted in migration inhibition comparable to that of knockdown of each gene individually. We then used the IncuCyte system to conduct scratch wound invasion assays on these cells, and noted that while all groups showed reduced scratch closure compared to the scrambled control, dual ABCE1 KD + LCP1 KD resulted in further reduction of scratch closure compared to ABCE1 KD or LCP1 KD alone (Figure 3B), suggesting an additive effect of ABCE1 and LCP1 on cell invasion. Next, we assessed the effect of ABCE1 KD, LCP1 KD, and ABCE1 KD + LCP1 KD on cell proliferation. Colony formation (Figure 3C) and anchorage-independent growth assays (Figure 3D) demonstrated decreased colonization of LCP1 KD cells compared to scrambled. Notably, dual ABCE1+ LCP1 downregulation resulted in additional reduction of cell proliferation compared to either ABCE1 KD or LCP1 KD alone.

### Dual knockdown of Abce1 and Lcp1 reduces tumor aggressiveness and prolongs survival *in vivo*

**Figure 4:**
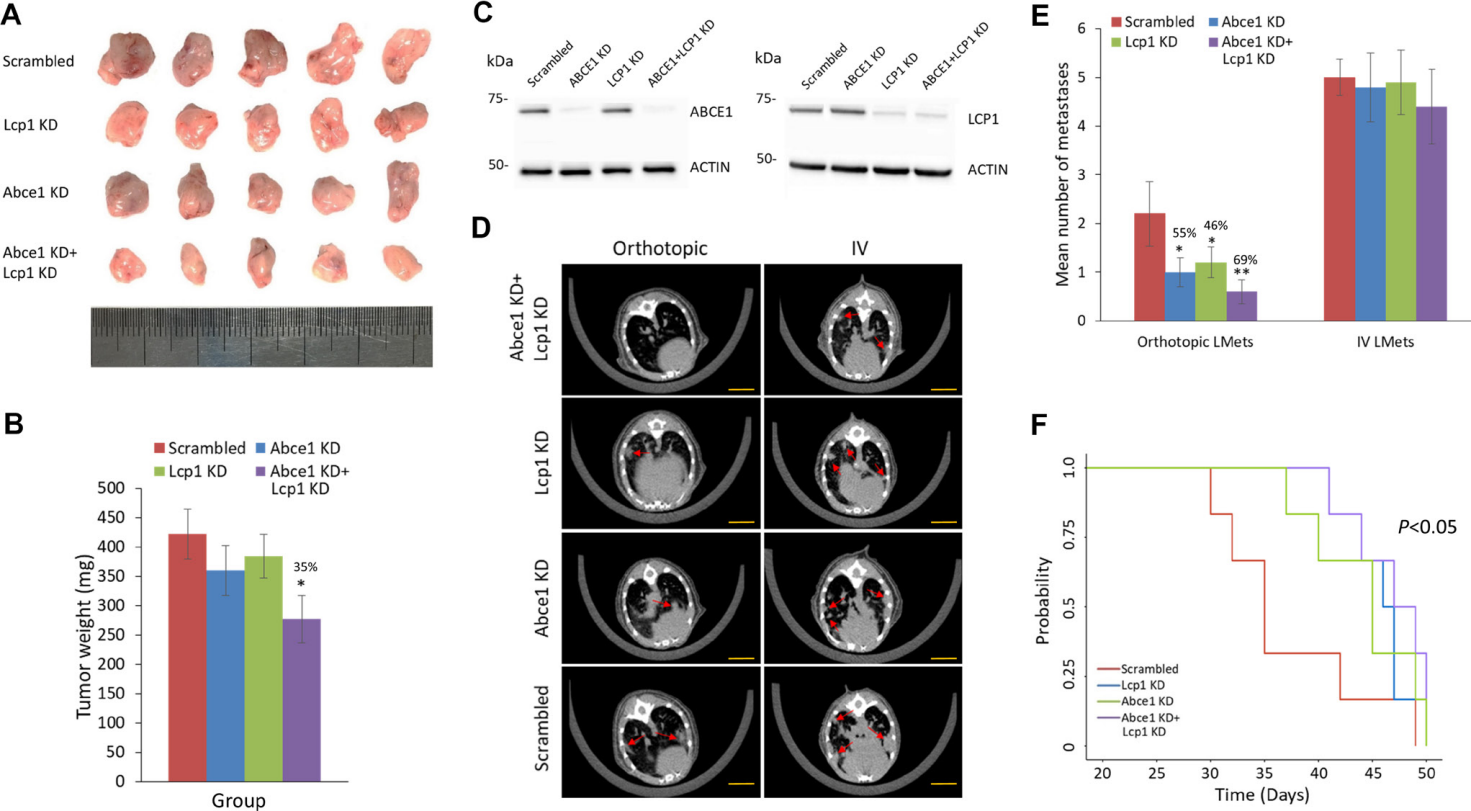
Reduced LCP1 expression in the early stages of metastasis decreases lung metastasis formation and is correlated with lower tumor grade and preferable intrinsic tumor subtype (**A**) Photographs of primary breast tumors of scrambled, Lcp1, Abce1 KD and Abce1 KD + Lcp1 KD 4T1 cells that were removed on day 14 post-injection. Ruler pictured in metric units. (**B**) Tumor weights were measured as they were harvested from the mice. Abce1 KD + Lcp1 KD tumors weighed significantly less than tumors of other treatment groups. (**C**) Western blot analysis of protein extracted from breast tumors derived from scrambled, Lcp1 KD, Abce1 KD, and Abce1 KD + Lcp1 KD 4T1 cells revealed reduced protein expression of Abce1 in Abce1 KD and dual Abce1+Lcp1 KD groups compared to the scrambled group, while the Lcp1 protein expression level was reduced in the Lcp1 KD and the dual Abce1+ Lcp1 KD groups compared to the scrambled group. (**D**) Lung microCT scans performed on day 28 (orthotopic) and day 21 (IV) post-4T1 injection and (**E**) quantification of LMets in microCT show significantly fewer metastatic growths (indicated by red arrows) in mice that orthotopically received in mice that orthotopically received Abce1 KD, Lcp1 KD or Abce1 KD + Lcp1 KD cells compared to the scrambled control. Injection of Abce1 KD+Lcp1 KD cells resulted in the most prominent reduction of orthotopic LMets compared to knockdown of Abce1 or Lcp1 alone. IV injection of Abce1 KD, Lcp1 KD or Abce1 KD + Lcp1 KD cells did not result in fewer lung foci compared to scrambled. Scale bars represent 1 mm. (**F**) Kaplan–Meier survival analysis demonstrated increased overall survival of mice that received Abce1 KD + Lcp1 KD compared to the scrambled control. Data are presented as mean ± SEM. ^*^p < 0.05.

After demonstrating the additive effect of LCP1 KD and ABCE1 KD *in vitro*, we analyzed the functional roles of dual ABCE1 and LCP1 inhibition on tumor growth, disease progression, and overall survival *in vivo*. To this end, Abce1 KD, Lcp1 KD, Abce1 KD+Lcp1 KD, or scrambled 4T1 cells were introduced orthotopically or intravenously into BALB/c female mice. Dual Abce1 KD+ Lcp1 KD significantly inhibited primary tumor growth compared to scrambled (Figure 4A, 4B) while Abce1 KD or Lcp1 KD alone did not reach statistical significance. RNA and protein expression levels of Lcp1 and Abce1 in the primary tumors were measured to validate the shRNAs effect (Figure 4C and Supplementary Figure 3). Remarkably, lung CT scans revealed a significant reduction in orthotopic-derived lung metastases (LMets) in all groups compared to scrambled (Figure 4D, 4E), with dual Abce1 KD+Lcp1 KD mice developing the lowest number of LMets. No significant difference in lung foci was observed when cells were introduced intravenously (Figure 4D, 4E). Lastly, when compared to scrambled cells, dual Abce1 KD+Lcp1 KD resulted in improved overall survival reaching statistical significance of *p* = 0.04 (Figure 4F). Knockdown of either Abce1 or Lcp1 alone appeared to improve overall survival, though not enough to reach statistical significance. Together, these results suggest that the additive effect of two miR-96 gene targets, ABCE1 and LCP1, has a functional role in the downregulation of breast cancer growth, aggressiveness, and survival.

## DISCUSSION

We recently demonstrated the onco-suppressive effect of miR-96 OE on breast cancer aggressiveness. Utilizing *in vitro* and *in vivo* models, we showed that ABCE1 acts as an oncogene and provided evidence of its direct downregulation by miR-96 OE [11]. As master regulators of gene expression, each miRNA potentially regulates multiple target genes [20]. In cellular pathways, the effect of a single miRNA-mRNA interaction might be minor, but the combined effect of a single miRNA on several target genes that act in the same biological pathway could significantly assist in altering cellular activity and phenotype [9]. In the current study, we filtered our previously published miR-96 gene target list by their involvement in cytoskeleton regulation and selected LCP1 for further investigation. We showed that LCP1 is downregulated in human and mouse breast cancer cell lines-both on the RNA and protein levels-in response to miR-96 overexpression. *In vivo* experiments corroborated these results, demonstrating a statistically significant mean decrease in Lcp1 RNA and protein expression in mouse primary tumors with miR-96 overexpression. Dual-Luciferase reporter assay confirmed the direct regulation of *LCP1* transcription by miR-96.

Human plastins are conserved throughout eukaryotic evolution and expressed in most tissues of higher eukaryotes [21]. LCP1, The L isoform of plastin, is a member of the actin-binding protein family. LCP1 is strongly expressed in hematopoietic cell lineages and is critical for many immune cell functions including formation of the immune synapse [22], migration, invasion [23], and adhesion [24] of hematopoietic cells. In addition, LCP1 has been found in nearly 70% of epithelial-derived cancers [21], suggesting that its expression contributes to metastatic capabilities of the transformed cells. Elevated LCP1 levels have been shown to correlate with increased cell migration and invasion in colorectal [25], oral [26], prostate [27], and cholangiocarcinoma [28] cancers, as well as with an increased number of lung metastases in breast cancer mouse models [29]. We demonstrated that downregulation of LCP1 resulted in significantly reduced breast cancer cell migration and invasion, and that dual ABCE1+LCP1 knockdown further inhibits cell motility, especially invasion. Likewise, decreasing LCP1 expression resulted in reduced anchorage dependent and independent growth, whereas simultaneous knockdown of both ABCE1 and LCP1 enhanced this effect. We also demonstrated that while dual ABCE1+ LCP1 downregulation did not affect cancer cell extravasation, it significantly decreased tumor growth and dissemination, and increased overall survival.

Detailed characterization of the relationship between ABCE1 and LCP1 has yet to be elucidated. Each gene is located on a different locus (ABCE1 on chr4 and LCP1 on chr13) and co-expression analysis does not reveal significant enrichment (data not shown), either by actin immunoprecipitation or by computational data-mining (gene-fusion and gene-network analyses). PubMed text-mining did not produce any studies that have explored the potential co-expression enrichment of ABCE1 and LCP1. Moreover, computational prediction did not reveal gene fusions or any other physical relationship between ABCE1 and LCP1 that can explain their additive role in cancer progression (data not shown). The metastatic process is highly complex, and involves numerous proteins acting simultaneously and spatially. Additional experiments need to be conducted to shed light on the ABCE1-LCP1 connection.

In conclusion, we demonstrated that LCP1 is directly regulated by miR-96 and has an important role in controlling breast cancer cell motility. We demonstrated that downregulation of LCP1 together with ABCE1 further reduces cell motility and proliferation *in vitro*, and overall survival *in vivo,* compared to ABCE1 or LCP1 alone. This coincides with the assumption that single miRNA regulates multiple genes and supports seeking more than one miRNA target in pre-clinical experiments in order to enhance functional effects.

## MATERIALS AND METHODS

### Orthotopic and IV breast cancer mouse models

Six-week-old female BALB/c mice were purchased from Envigo RMS Laboratories (Ness Ziona, Israel). Mice were maintained according to the guidelines of the Tel Aviv University Institutional Animal Care and Use Committee. Each study group consisted of five mice. Orthotopic tumors were induced by exposing the fourth (inguinal) mammary fat pad and injecting it with 2 × 10^5^ 4T1 cells suspended in 50 μL of PBS (Biological Industries). The IV metastatic breast model was achieved by tail vein injection of 10^4^ 4T1 cells suspended in 100 μL of PBS (Biological Industries). Tumor growth was assessed by measuring individual tumors with calipers and calculating tumor volume: Tumor volume (mm^3^) = (width × length^2^) / 2. Mean final tumor weight and mean number of metastases were compared between groups using the Fisher Exact test.

### Cell lines and expression regulation

Breast cancer cell lines (4T1, MDA-231, and HS578), HeLa cells, and HEK-293T cells were described previously [30]. Cells were cultured in Dulbecco’s Modified Eagle’s Medium (Biological Industries) supplemented with 10% fetal bovine serum (GIBCO). Before use, each cell line was confirmed to have no mycoplasma contamination using the EZ-PCR Mycoplasma Test Kit (Biological Industries). Short hairpin RNAs (shRNAs) for LCP1 and scrambled vector were established as described previously [31]. Transient and stable cell lines expressing scrambled control miRNA or miRNA-96 overexpression were established as described previously [32]. Plasmids pLKO.1-LCP1, pLKO.1-ABCE1, and pLKO.1-scrambled were purchased from Dharmacon. pLKO.1-Double (expressing LCP1 and ABCE1 shRNAs) was created as described previously [33], using half of the ABCE1 and LCP1 shRNAs, reaching a total shRNA amount equivalent to that of the plasmids expressing single shRNAs.

### RNA analysis

Total RNA extraction and reverse transcription were performed as previously described [34]. mRNA was reverse transcribed with random primers and SuperScriptIII reverse transcriptase (Thermo Fisher). mRNA expression was tested similarly using SYBR green PCR master mix (Thermo Fisher) by means of the StepOnePlus real-time PCR system (Thermo Fisher). Specific primer pairs for mRNA expression detection were ordered from IDT (Supplementary Table 1). Expression values were calculated based on the comparative threshold cycle method. Expression levels were normalized to human GAPDH or mouse actin.

### IncuCyte live cell imaging system

The IncuCyte S3 system and software (Sartorius) were used for migration and invasion assays according to the manufacturer’s protocols. In 96-well plates (IncuCyte ImageLock Plates 4379), 2 × 10^4^ 4T1 cells/well were seeded for each assay. Images were analyzed with the IncuCyte HD software (Sartorius) and the results presented as relative wound densities and standard deviations for each time point. Relative wound density (%) represents the cell density in the scratch area relative to that outside the scratch, as a function of elapsed time. Experiments were performed in replicates of five per condition.

### Dual luciferase reporter assay

The predicted binding site for miR-96 on the 3′UTR of LCP1 was PCR-amplified as previously described [35] and cloned into the psiCHECK-2 plasmid (Promega). Negative control of LCP1 was achieved by substituting 3 nucleotides in the seed binding region of the cloned 3′UTRs using the QuikChange Lightning SDM kit (Agilent), as previously described [36]. HEK-293T and HeLa cells were seeded in 24-well plates supplemented with 10% FBS (GIBCO). Cells were transfected using Lipofectamine 2000 (Rhenium), 5 ng of the psiCHECK-2 relevant clone, 10 ng of pEGFP, and 485 ng miRVec containing the desired pre-miRNA. Twenty-four hours following transfection, lysates were extracted and firefly and Renilla luciferase activities were measured using the Dual-Luciferase Reporter Assay System Kit (Promega). The Renilla luciferase results were normalized to the values of the firefly luciferase.

### Colony formation assay

HS578 and 4T1 cells (scrambled/miR-96 OE/ABCE1 KD/miR-96 OE+ABCE1 OE) were seeded in 6-well plates (1 × 10^3^ cells per plate) and cultured for 8 days (4T1) or 14 days (HS578). The colonies were fixed and stained with 0.5% crystal violet mixed with 10% methanol for 30 min. Quantification of stained, fixed colonies was done using ImageJ software (NIH). All experiments were performed in triplicate.

### Anchorage-independent growth ability assay

1 × 10^3^ 4T1 or HS578 cells (scrambled/miR-96 OE/ABCE1 KD/miR-96 OE+ABCE1 OE) were detached by Trypsin and resuspended in 2 ml complete medium plus 0.33% noble agar (Invitrogen). The mixture was seeded in a six-well plate containing 0.66% complete medium/noble agar mixture. Colony sizes were measured with an ocular micrometer after 13 days (4T1) or 23 days (HS578) of incubation and colonies greater than 0.1 mm in diameter were scored. All experiments were performed in triplicate.

### Western blot analysis

Cells were homogenized with lysis buffer containing 50 mM Tris HCl (pH 7.6), 20 mM MgCl_2_, 150 mM NaCl, 0.5% NP40, and 5 units/mL Aprotinin (Sigma-Aldrich). Lysates were collected after centrifugation and protein concentrations determined using the Bio-Rad protein assay (Bio-Rad Laboratories). Lysates were resolved by SDS–polyacrylamide gel electrophoresis (SDS-PAGE) using 4–12% gels (Gentaur), and electrophoretically transferred to a nitrocellulose membrane. Membranes were blocked for 1-2 hours in TBST buffer (0.02 M Tris HCl pH 7.5, 0.15 M NaCl, and 0.05% Tween 20) containing 5% milk, and then incubated with dilute primary antibody (Supplementary Table 2) in blocking solution overnight at 4° C. Membranes were washed in TBST buffer 3 times, and incubated with a secondary antibody (Supplementary Table 3) for 45 minutes at room temperature. Immunoreactive bands were detected with enhanced chemiluminescence reagent (Thermo Fisher) and quantified using ImageJ software (NIH).

## SUPPLEMENTARY MATERIALS FIGURES AND TABLES


