# Supplementary material for: Dual inhibition of ABCE1 and LCP1 by microRNA-96 results in an additive effect in breast cancer mouse model
Source: Oncotarget. 2019 Mar 12;10(21):2086–94. doi: 10.18632/oncotarget.26747 (PMC6459344; doi:10.18632/oncotarget.26747)
Supplement: Supplementary file 1 [file oncotarget-10-2086-s001.pdf]

Dual inhibition of ABCE1 and LCP1 by microRNA-96 results in an additive effect in breast cancer mouse model

SUPPLEMENTARY MATERIALS

Supplementary Table 1: SYBR green RT-PCR primers for mRNA quantification

Primer name	Sequence
M Lcp1 For	TCTCACTGTCACGGTGGTTGGC
M Lcp1 Rev	CTCAAATCTCCAGAGAAGCAAGCAC
H Lcp1 For	CGTTGGCACCCAACACTCCTA
H Lcp1 Rev	GGGATGACATGCCGACAATCAG
M β-Actin-For	ACCAGAGGCATACAGGGACA
M β-Actin-Rev	CTAAGGCCAACCGTGAAAAG
H GAPDH-For	AGCCACATCGCTGAGACA
H GAPDH-Rev	GCCCAATACGACCAAATCC

Abbreviations: *H-human, M-mouse.

Supplementary Table 2: Primary antibody used for Western blot analysis

Antibody	Manufacturer	catalog number	Dilution
Mouse anti-LCP1	Santa Cruz	sc-133219	1:1000
Mouse anti-Actin	Millipore	clone C4, MAB1501	1:5000

Supplementary Table 3: Secondary antibody used for Western blot analysis

Antibody	Manufacturer	Catalog number	Dilution
HRP-conjugated AffiniPure Goat-anti-Mouse IgG	Jackson ImmunoResearch Laboratories, Inc.	115-035-166	1:10000

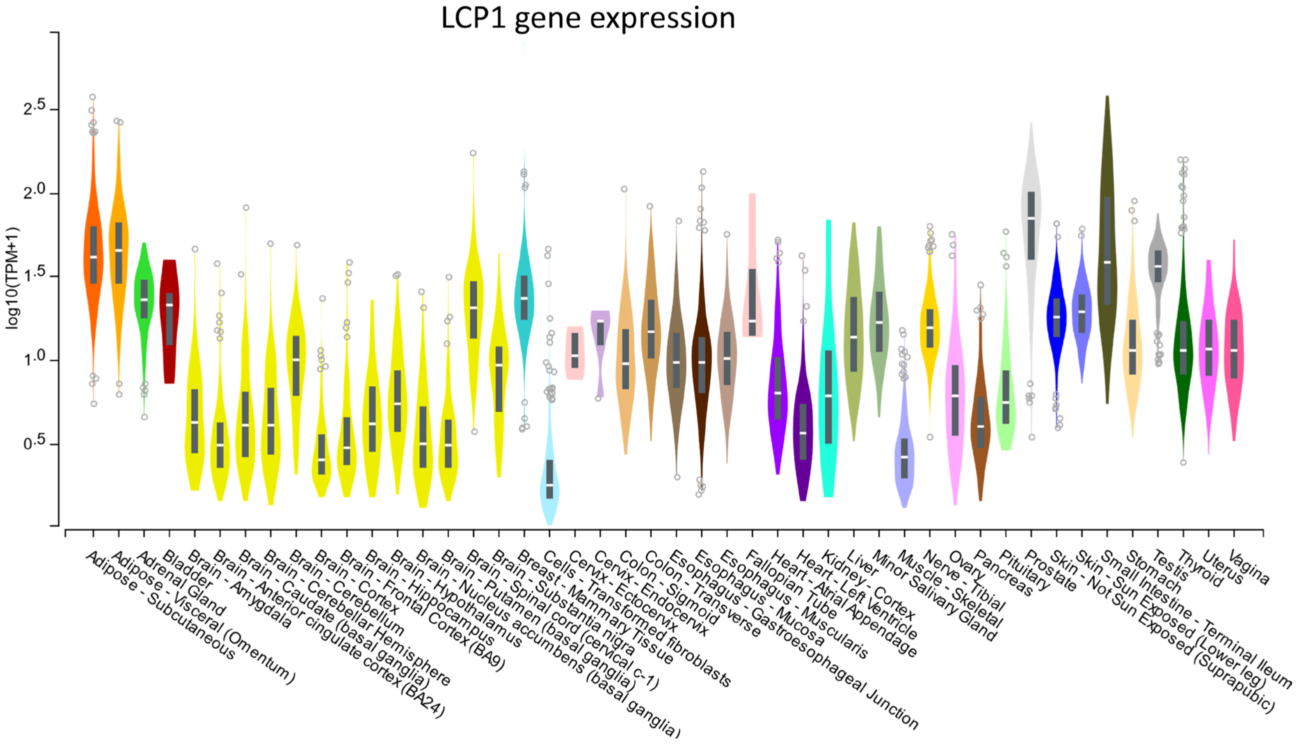

Supplementary Figure 1: LCP1 mRNA expression in 53 non-diseased tissue sites. LCP1 mRNA expression levels were determined in 53 non-diseased tissue sites collected from nearly 1,000 individuals using data from the GTEx project [19]. TPM- transcripts per million kilobases.

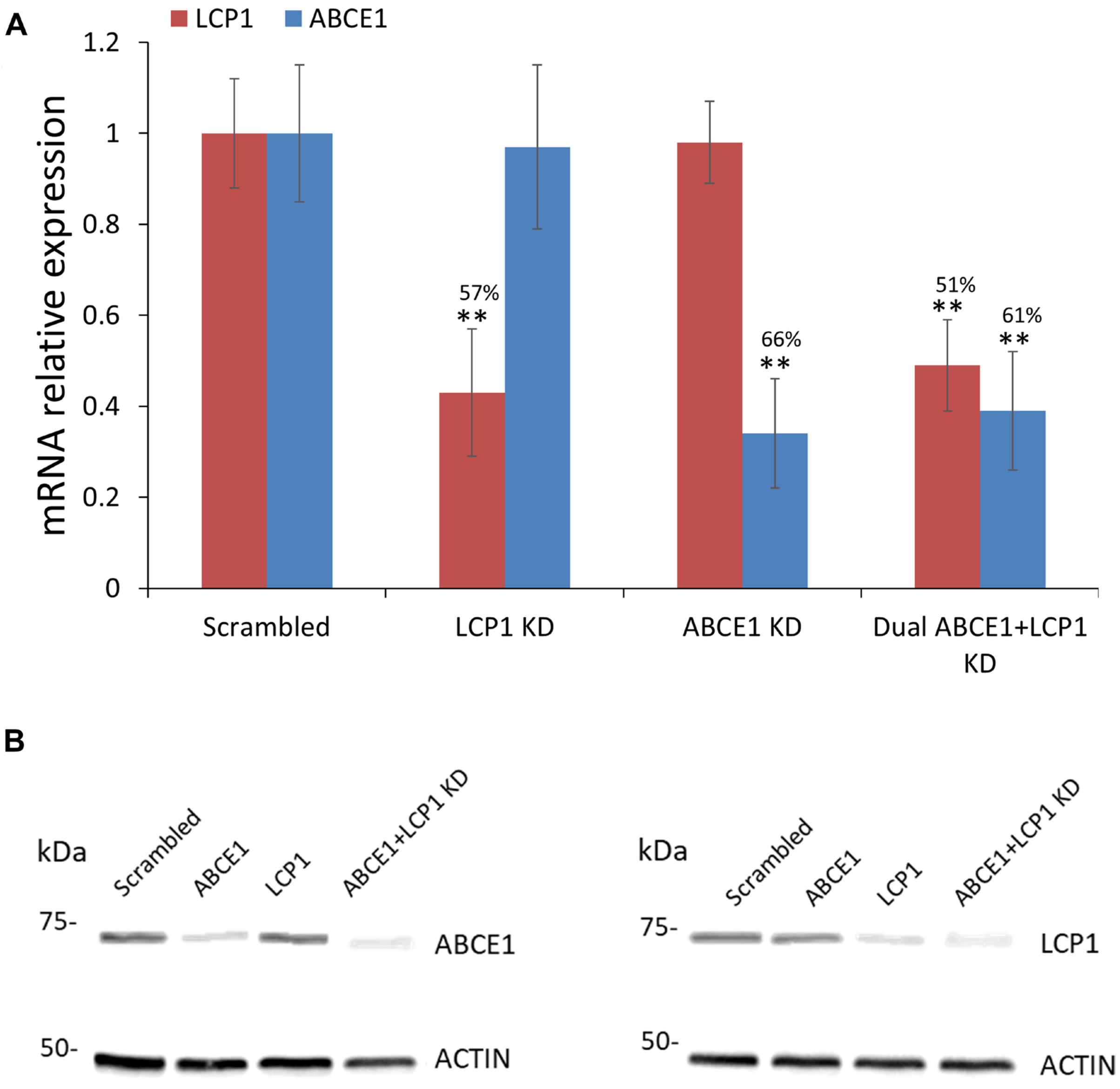

Supplementary Figure 2: LCP1 and ABCE1 mRNA and protein expression in cells that stably underexpress Abce1, Lcp1, Abce1+Lcp1, or scrambled. (**A**) Expression levels of ABCE1 and LCP1 were calculated for 4T1 cells stably transfected with shRNAs against Abce1/ Lcp1/Abce1+ Lcp1/ scrambled. ABCE1 RNA expression was reduced in ABCE1 KD and ABCE1+LCP1 KD tumors, while LCP1 was decreased in LCP1 KD and ABCE1+LCP1 derived tumors. (**B**) Western blot analysis of protein extracted from the cells in (A) revealed reduced protein expression of Abce1 in Abce1 KD and dual Abce1+Lcp1 KD groups compared to the scrambled group, while the Lcp1 protein expression level was reduced in the Lcp1 KD and the dual Abce1+ Lcp1 KD groups compared to the scrambled group. Data is presented as mean ± SEM. $^{**}p < 0.01$.

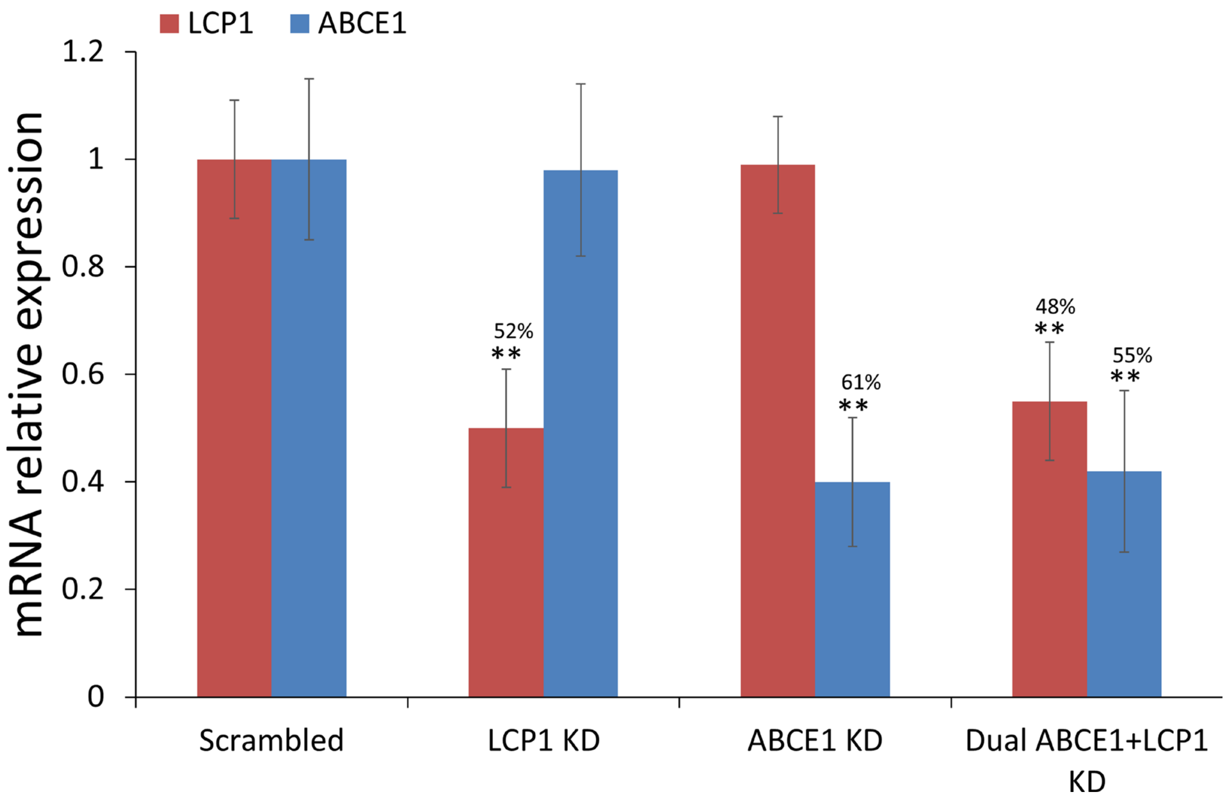

Supplementary Figure 3: LCP1 and ABCE1 mRNA expression in mouse tumors. Expression levels of ABCE1 and LCP1 were calculated for breast tumors originating from Abce1 KD, Lcp1 KD, Abce1 KD+Lcp1 KD, or scrambled 4T1 cells. ABCE1 RNA expression was reduced in ABCE1 KD and ABCE1+LCP1 KD tumors, while LCP1 was decreased in LCP1 KD and ABCE1+LCP1 derived tumors. Data is presented as mean ± SEM. $^{**}p < 0.01$.
